# Resolution of childhood wheat allergy through evolution into WALDA? A case report 

**DOI:** 10.5414/ALX02631E

**Published:** 2026-06-16

**Authors:** Charlotte J. Kiani, Valentina Faihs, Claudia Kugler, Julia F. Pilz, Tilo Biedermann, Knut Brockow

**Affiliations:** 1 Department of Dermatology and Allergy, Technical University of Munich, TUM School of Medicine and Health, Munich, Germany, and; 2 Department of Dermatology and Allergy Centre, Odense University Hospital, Odense, Denmark

**Keywords:** WALDA, wheat allergy, WDEIA, anaphylaxis, augmentation factor

## Abstract

Background: Wheat allergy may present with different phenotypes. Childhood-onset wheat allergy is characterized by immediate-type IgE-mediated reactions to wheat ingestion alone. It typically affects atopic individuals and often resolves spontaneously. The predominant adult-onset phenotype is WALDA (wheat allergy dependent on augmentation factors), triggered only when wheat is consumed in combination with augmentation factors. Phenotypic transition between these forms is rarely described. Case report: We present the case of a 30-year-old atopic female patient with a history of wheat-induced anaphylaxis, who presented with three distinct stages, compatible with a phenotypic transition. In infancy, the patient was diagnosed with classical IgE-mediated wheat allergy. In early adulthood, she developed augmentation factor-dependent reactions compatible with WALDA. At the age of 30, comprehensive oral food challenge testing and serological analysis revealed full clinical tolerance and loss of sensitization. Conclusion: This case illustrates a possible transient clinical course of wheat allergy with WALDA as an intermediate stage prior to resolution. Such cases may be underreported, as patients with declining wheat allergy may not be identified as having WALDA reactions, but rather as having inconstant reactivity. Allergy reassessment in adult patients with food allergy in childhood is essential to detect phenotypic shifts or to confirm resolution.

## Introduction 

Wheat allergy may present with different phenotypes. Classical IgE-mediated wheat allergy typically develops in early childhood, particularly in children with atopic eczema and often resolves spontaneously [1]. In contrast, wheat allergy dependent on augmentation factors* (WALDA) is the predominant clinical manifestation of adult-onset wheat allergy [2]. In WALDA, augmentation factors such as physical exercise, intake of non-steroidal anti-inflammatory drugs, or alcohol are needed to elicit allergic reactions [3]. We do not know of a reported case in the literature, where allergy resolved with WALDA as an intermediate stage, and speculate that such a course may be underreported. Here, we report the case of a 30-year-old female patient with a pronounced atopic background (including atopic dermatitis, bronchial asthma and allergic rhinitis), who presented with an unequivocal history of wheat-associated anaphylaxis showing phenotypic changes over time, ultimately leading to complete resolution. 

*Annotation: The term WDEIA (wheat-dependent exercise-induced anaphylaxis) has been historically used to describe wheat anaphylaxis occurring in conjunction with exercise as an augmentation factor. However, WDEIA does not accurately include other possible augmentation factors such as alcohol or nonsteroidal anti-inflammatory drugs. Thus, the usage of the term WALDA (wheat allergy dependent on augmentation factors) is considered a more accurate and comprehensive terminology [15]. 

## Case report 

In early childhood, the patient was diagnosed with “classical” IgE-mediated wheat allergy. At the age of 1 year, she experienced her first episode of anaphylaxis following the ingestion of a small amount of pasta. Clinical symptoms included apathy and cyanotic lips, indicative of systemic hypotension and respiratory compromise. Three additional anaphylactic episodes occurred at the age of 1, 2, and 4 years, each triggered by accidental ingestion of small amounts of wheat-containing foods such as pretzel and pasta. These episodes were again characterized by apathy and cyanosis, and in one case, additional wheezing. In each instance, immediate antiallergic treatment and hospitalization were required. Based on these reactions, the patient strictly avoided wheat consumption. Given the absence of symptoms during this period, she cautiously reintroduced wheat at the age of 15, with very low and slowly increasing amounts of wheat products and initially without any noticed symptoms. Approximately at the age of 20, the patient reported frequent (10 – 15) allergic reactions with generalized urticaria, angioedema, gastrointestinal symptoms, and tachycardia when wheat ingestion was followed by physical activity or alcohol consumption. The plausible clinical history, combined with low-level sensitization to wheat (1.0 kU/L) and gliadin (0.7 kU/L) as well as positive skin prick test to wheat (++), led to the diagnosis of WALDA in 2016 by her treating outpatient allergist at that time, although specific IgE to ω-5-gliadin as predominant allergen in WALDA was negative. As a result, the patient began avoiding wheat together with known augmentation factors. While she continued to consume wheat products in the evenings, she was careful to ensure a sufficient time gap of at least 4 – 6 hours before physical activity. Under this modified behavior, only rare accidental reactions occurred, supporting the suspected diagnosis of WALDA. The last reported event developed at the age of 29 years after she consumed a pizza followed by a walk and subsequently experienced a reaction with generalized urticaria, angioedema, and tachycardia, which subsided after combined antiallergic treatment with antihistamines and corticosteroids. 

One year later, at the age of 30 years, the patient presented to our department, aiming for more individualized dietary counselling and optimization of disease management. Thus, we decided for an oral food challenge test and repeated specific IgE analysis [3]. Specific IgE levels were measured using ImmunoCAP (Thermo Fisher, Uppsala, Sweden) and ALEX2 (Macro Array Diagnostics, Vienna, Austria). Neither specific IgE to gliadin (< 0.1 /L) nor to ω-5-gliadin (Tri a 19; < 0.1 kU/L) was detectable, despite a total IgE concentration of 309 kU/L. Specific IgE to other cereal components including barley (0.1 kU/L), oat (0.2 kU/L), rye (0.3 kU/L), gluten (0.1 kU/L), wheat flour (0.4 kU/L), and wheat lipid transfer protein Tri a 14 (< 0.1 kU/L) were all low or negative. In the oral food challenge, the patient tolerated not only 8, 16, and 32 g of pure wheat gluten, but also 8, 16, and 32 g of wheat gluten in combination with 1,000 mg of acetylsalicylic acid (ASA) as well as 32 g of wheat gluten in combination with 1,000 mg of ASA, 20 mL of 95% alcohol (Braun, Melsungen, Germany), and 20 minutes of intensive running on a treadmill according to our protocol [4]. Because of very high doses of wheat allergen given in combination with several augmentation factors, this protocol has demonstrated a near 100% sensitivity for WALDA [4]. Taken all findings together, there is currently no objective evidence for wheat allergy or WALDA left in the patient. Thus, wheat allergy in this patient has resolved after 3 decades and she has been advised to continue regular wheat intake without avoiding any augmentation factors in conjunction.[Table Table1]

## Discussion 

This case report suggests that both, WALDA and classical wheat allergy do not only share the immunological IgE-mediated mechanism but may also sequentially occur in the same patient. A similar pattern has already been described for cow’s milk. A 16-year-old girl with a history of primary cow´s milk allergy in childhood was reported to tolerate milk when ingested alone, but to develop anaphylactic symptoms when milk consumption was combined with physical exercise, which was confirmed by oral food challenge [5]. 

**Figure 1.**
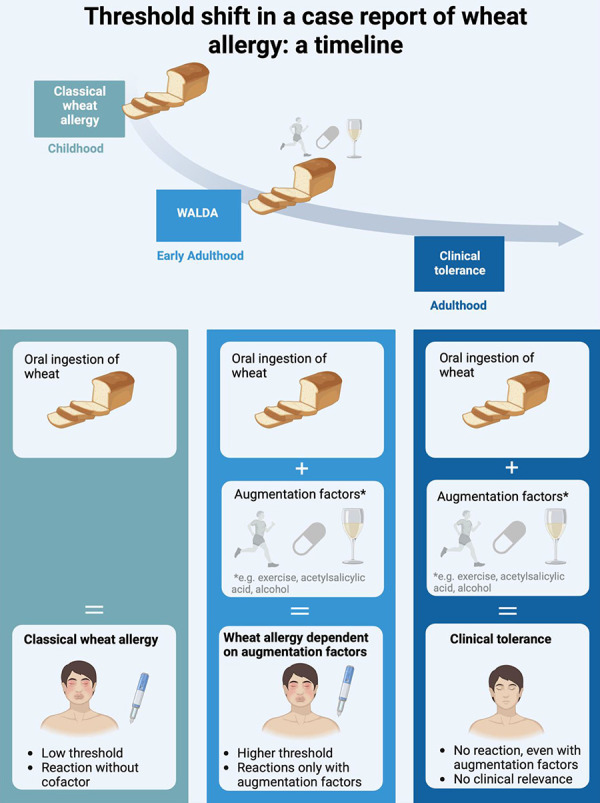
Phenotypic evolution of the patient’s wheat allergy from classical childhood allergy to WALDA and finally tolerance. Created in BioRender. Köberle, M. (2025) https://BioRender.com/lxzebq8.

Nevertheless, there are important differences in both phenotypes, not only in their reaction threshold and thus the need of additional augmentation factors increasing the absorption (Figure 1). In classical wheat allergy, reactions occur upon ingestion alone, as demonstrated in this patient’s anaphylaxis in early childhood. Later, with decreasing sensitization, WALDA may have developed requiring augmentation factors such as exercise, nonsteroidal anti-inflammatory drugs, or alcohol to elicit an allergic reaction [3]. 

The prognosis may differ between classical wheat allergy and WALDA. Most children with wheat allergy outgrow the allergy as they get older. Tolerance to wheat was reported to develop in 96% of patients at 16 – 18 years of age [6]. In contrast, a study where ω-5-gliadin IgE was measured at least twice in 63 ω-5-gliadin-positive and -dominant WALDA patients over an observation period of ≥ 24 months (range 24 – 69 months) showed that in the majority (> 85%) of patients ω-5-gliadin IgE did not decrease over time during several years of observation. Furthermore, only 1 patient showed a clinical remission implying a poor prognosis for this phenotype [7]. This is in accordance with our own observation that regression in our WALDA patients appears uncommon. A limitation of the data on WALDA, however, is that the observation period in these patients generally has been a few years in comparison to 1 – 2 decades in classical wheat allergy. 

Although ω-5-gliadin is considered the predominant allergen in WALDA, specific IgE to this component was negative in our patient. Low-level sensitization to wheat or other gliadin components, in conjunction with a compatible clinical history, may still support the diagnosis of WALDA, as it has been already described for rare cases of grass pollen-related WALDA and patients sensitized through the percutaneous use of hydrolyzed wheat proteins [11, 12, 13]. These findings not only reflect phenotypic variability but may emphasize underlying immunological heterogeneity within the WALDA spectrum. 

Interestingly, patients with WALDA typically report no preceding childhood wheat allergy. However, the described patient presented unequivocal histories of wheat allergy and WALDA, which were confirmed by her pediatrician and allergist. In the absence of allergic reactions in later childhood and without an oral food challenge at the time of the reported augmentation-factor dependent episodes, it cannot be excluded that first wheat allergy and then WALDA occurred and then resolved independently. However, this appears to be an unlikely coincidence, which has not been described before. The clinical course is more consistent with a evolution from wheat allergy to WALDA prior to resolution. Similarly, during oral immunotherapy with foods, patients may develop reactions when augmentation factors are present at the time of the food intake [8, 9]. Furthermore, a case series in patients after successful oral immunotherapy (OIT) for wheat and cow’s milk allergy reported allergic reactions to receiving the maximum desensitization dose followed by exercise. Whereas specific IgE levels were not discriminative, older age and longer time to achieve the full dose were described as risk factors for exercise-induced reactions [10]. 

Taken together, this case may represent an unusual clinical pattern, in which WALDA appeared to emerge as an intermediate entity in the patient’s gradual decline in clinical reactivity to wheat tolerance over the years. Natural resolution of childhood wheat allergy and independent development of WALDA cannot be definitively excluded, but appears unlikely. Furthermore, it is conceivable that such patients are underreported, because in patients with declining wheat allergy, WALDA reactions may not be recognized as a new phenotype, rather as an inconstant reactivity. Finally, this case highlights the importance of allergy re-evaluation in adult patients with wheat allergy, as resolution or changes in clinical reactivity with time are likely to occur [14]. 

## Acknowledgment 

We would like to thank Martin Köberle for creating the figure using BioRender.com. 

## Authors’ contributions 

Conceptualization: CJK, VF, KB; Data curation: VF, KB; Funding acquisition/resources: TB, KB; Investigation: VF, CK, KB; Methodology: CJK, VF, KB; Project administration: CJK, KB; Supervision: TB, KB. Visualization: CK; Writing – Original draft preparation: CJK, VF, KB; Writing – review & editing: CJK, VF, CK, JFP, TB, KB. 

## Funding 

This work was supported by the German Federal Ministry of Education and Research (Bundesministerium für Bildung und Forschung, BMBF) as part of the ABROGATE project (grant number 01EA2106A) awarded to KB and by the Clinician Scientist Program TRIAL of the German Society for Allergy and Clinical Immunology (DGAKI) to VF. Additional support was provided by the German Research Foundation (Deutsche Forschungsgemeinschaft, DFG) within the RTG 2668 framework to KB and TB. 

## Conflict of interest 

The authors report no conflicts of interest related to this work. 

**Table 1. Table1:** Molecular sensitization profile including total IgE and specific IgE levels: Comparison between WALDA diagnosis and clinical tolerance.

**Total and sIgE ImmunoCAP** *^a^* **, kU/L**	**WALDA diagnosis (2016)**	**Clinical tolerance (2025)**
Total IgE	ND	309
sIgE ω-5-gliadin (Tri a 19)	< 0.1	< 0.1
sIgE LTP (Tri a 14)	< 0.1	< 0.1
sIgE Gliadin	0.7	< 0.1
sIgE Gluten	ND	0.1
sIgE Wheat	1.0	0.4
sIgE Barley	ND	0.1
sIgE Oat	ND	0.2

sIgE = specific IgE; ND = not done. ^a^Phadia. IgE values < 0.1 kU/L were considered negative.
